# A Rare Encounter of Postoperative Abscess Not Linked to Staple Line in Sleeve Gastrectomy: A Case Report and Literature Review

**DOI:** 10.7759/cureus.69813

**Published:** 2024-09-20

**Authors:** Yesenia Brito, Jonathan Valdivia, Ana I Gonzalez, Henry C Valdivia, Frederick Tiesenga, Juaquito Jorge

**Affiliations:** 1 Surgery, St. George's University School of Medicine, True Blue, GRD; 2 Pediatrics, St. George's University School of Medicine, True Blue, GRD; 3 Medicine, St. George's University School of Medicine, True Blue, GRD; 4 General Surgery, West Suburban Medical Center, Chicago, USA; 5 General and Bariatric Surgery, Tiesenga Surgical Associates, Elmwood Park, USA

**Keywords:** abdominal wall abscess, acute kidney injury(aki)), laparoscopic sleeve gastrectomy complications, postoperative abscess, streptococcus anginosus, surgical abdominal exploration

## Abstract

Sleeve gastrectomy is a common bariatric procedure known for its safety and effectiveness, but postoperative complications like abscess formation, though rare, can occur. We report the case of a 37-year-old female who presented with atypical abdominal pain following a sleeve gastrectomy. Imaging revealed an abscess located away from the staple line. Surgical exploration and culture identified *Streptococcus anginosus* as the causative organism. This case emphasizes the importance of vigilant postoperative monitoring and early intervention to prevent complications. Proper management, including antibiotics and surgical drainage, is crucial for patient recovery.

## Introduction

Sleeve gastrectomy is a widely used bariatric surgical procedure, preferred over other approaches due to its lack of anastomosis, which reduces the risk of complications such as fistula or stricture formation [1]. This surgery is recommended for individuals with a class one obesity body mass index (BMI) of <35 kg/m^2^ who have not achieved weight loss through lifestyle modifications [2]. For those with a BMI of >35 kg/m^2^, bariatric surgery is generally recommended, regardless of the presence of obesity-related comorbidities [2]. Studies indicate that sleeve gastrectomy significantly improves quality of life and overall health, outweighing the risks of perioperative complications [2].

The mortality rate for sleeve gastrectomy is under 3% and as low as 0.12% in patients with low perioperative risk factors [1]. Several variables, including age, gender, preoperative BMI, and underlying medical conditions, influence the likelihood of complications, which can be categorized as early (within 30 days post-surgery) or late (after 30 days) [3].

Early complications include bleeding, gastric leaks, and intra-abdominal abscesses, while late complications may involve gastric stenosis, nutrient deficiencies, and hernia formation [3]. Intra-abdominal abscess formation, a potential early complication, can present with postoperative fever and abdominal pain [4]. Abscesses following sleeve gastrectomy have been documented near the surgical site and on the spleen [5-7].

Prompt recognition of complications through frequent post-surgical follow-up visits, particularly within the first month, is crucial for timely intervention and resolution [4]. Overall, sleeve gastrectomy offers substantial benefits for patients with obesity and its comorbidities. Raising awareness surrounding the potential complications is essential for early detection and management. Placing an emphasis on postoperative care, including dietary guidance to prevent micronutrient and vitamin deficiencies, plays a vital role in the long-term success of the procedure [8].

## Case presentation

A 37-year-old female presented to the emergency department (ED) with complaints of left middle-sided abdominal pain for the past seven days. The patient stated the pain has been intermittent and progressively worsening. The pain was exacerbated with certain movements, such as walking, sitting, and shifting in bed. The patient states she has been having chills and fatigue. She denies nausea, vomiting, fever, dizziness, chest pain, and shortness of breath. 

Seven days prior to the ED visit, the patient underwent elective sleeve gastrectomy. After the procedure, the patient recovered appropriately and was discharged home two days later. 

The patient’s past medical history is significant for morbid obesity, prediabetes (not on medications), anxiety, nightmares, and hypovitaminosis D. She is a former cigarette smoker with a history of two packs per day for 15 years. She currently uses vapes and denies alcohol and illicit drug use. 

Upon assessment, the patient presented with a temperature of 99°F (37.2°C) and mild tachycardia with a pulse of 100 beats per minute (bpm) and a respiratory rate of 20 breaths per minute. The patient’s blood pressure was measured at 118/78 mmHg. Oxygen saturation levels were within normal limits, measuring 96% on room air. BMI was 51.16 kg/m^2^. The patient was alert, cooperative, anxious, and fatigued. Abdominal examination revealed left middle quadrant and left lower quadrant tenderness, positive bowel sounds, and clean, dry, and intact surgical sites. Lower extremities were equal bilaterally, with no edema or tenderness in the calves or thighs. 

**Figure 1:**
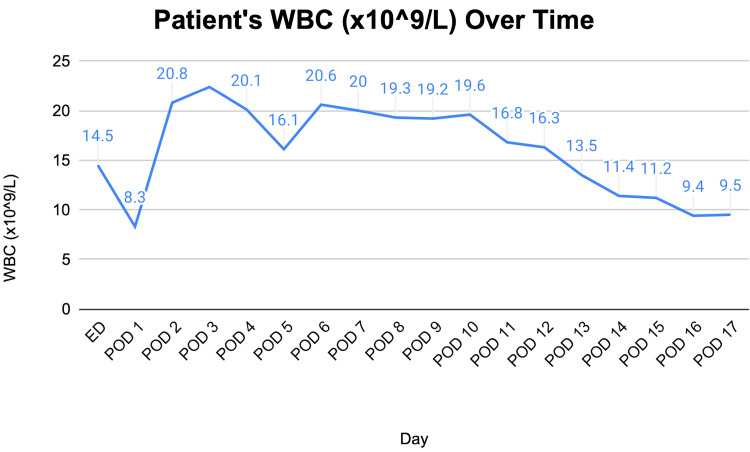
Line graph showing the WBC trend over time

**Figure 2:**
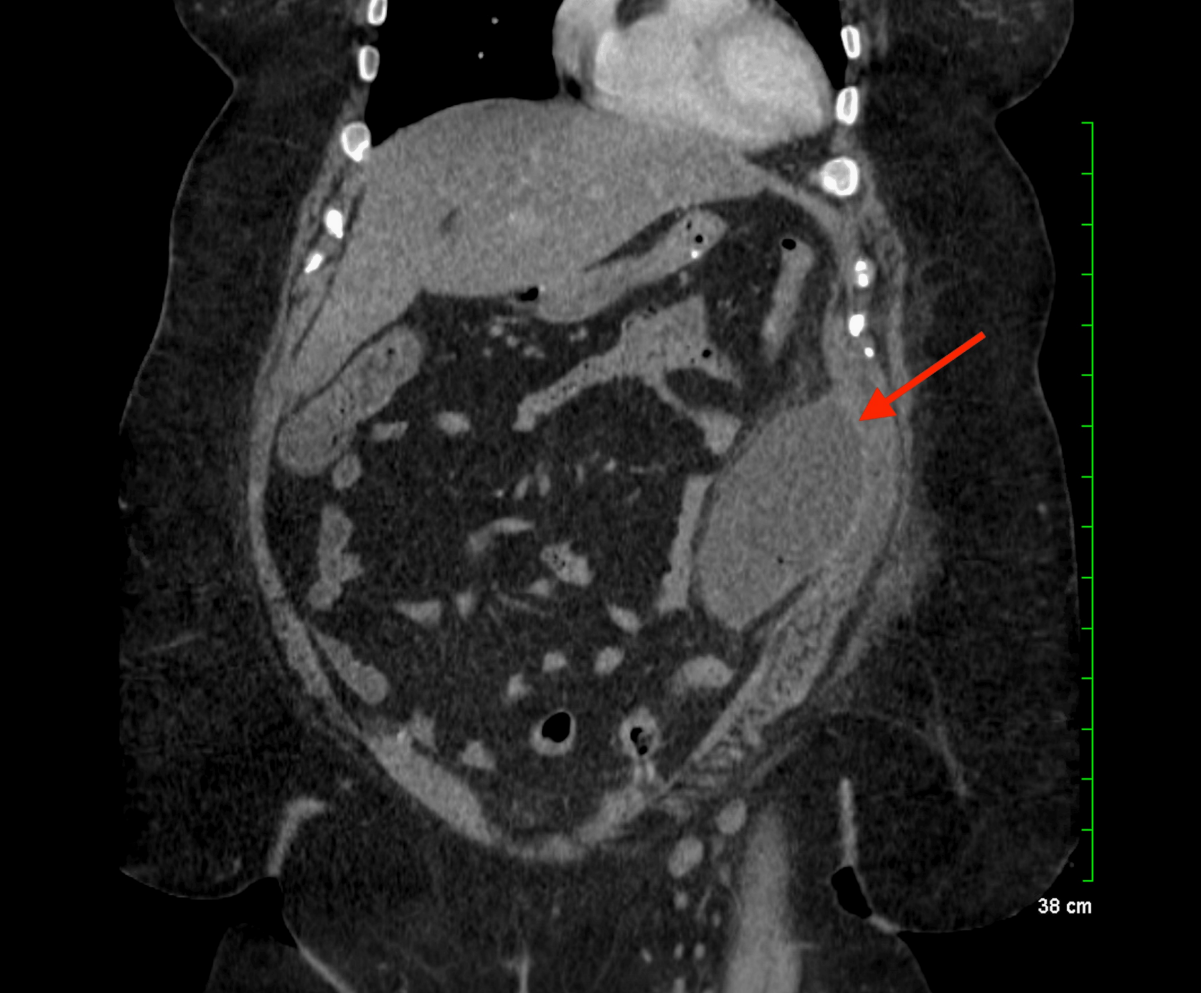
Ovoid abdominal fluid collection measuring 7.9 × 6.3 × 13.1 cm in greatest AP by transverse by craniocaudal dimensions with mild adjacent fat stranding concerning abscess Collection abuts the left anterior abdominal wall musculature.

The patient’s laboratory tests revealed a white blood cell (WBC) count of 14.5 (Figure 1) compared to 14 on postoperative day (POD) 1 status post-sleeve gastrectomy, and a lactate level of 0.9. A computed tomography (CT) scan of the abdomen and pelvis with intravenous (IV) contrast showed an ovoid intra-abdominal wall fluid collection concerning an abscess (Figure 2). The patient was started on antibiotics: vancomycin and piperacillin-tazobactam. The case was discussed with general surgery, and the patient was taken to the operative room (OR) for definitive management.

In the OR, the patient underwent laparoscopic exploration. A fair amount of adhesions were visualized on the left middle abdomen. Adhesions were released, and the omentum, which was adhered to the left mid-abdominal wall (far away from the site of the sleeve) at the site of the patient’s tenderness, was dissected. Upon opening this area, a large volume of foul-smelling fluid was encountered. Cultures were taken, and the area was irrigated with antibiotics. Although there was no obvious connection to the colon, the abscess was very close to the left colon, approximately at the level of the mid-descending colon. The rest of the exploration revealed no remarkable findings; the gastric sleeve appeared. The surgical wounds were closed in an anatomical fashion, and the procedure was completed.

On POD 2 status post-intra-abdominal wall abscess exploration, the patient’s initial leukocytosis had resolved. However, the empiric antibiotic regimen was continued pending the outcomes of blood and operative cultures.

**Figure 3:**
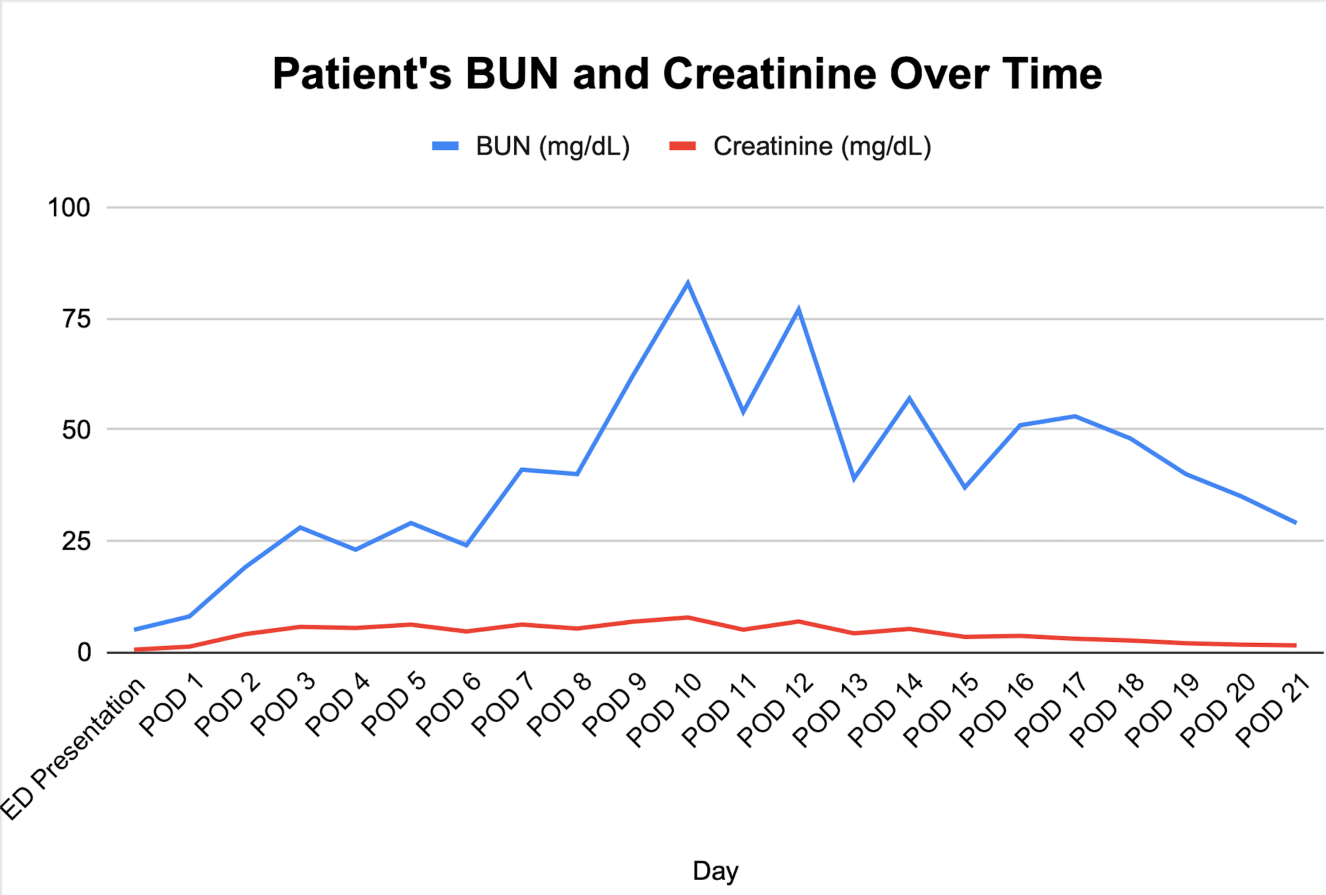
Line graph showing BUN and Creatinine trend over time

On the third day, the nephrology team was consulted due to acute kidney injury (AKI) and oliguria, marked by a rapid rise in the patient’s creatinine to 4.01 (Figure 3), reduced albumin levels, and poor urine output. However, potassium and bicarbonate levels were stable. The patient’s WBC increased to 20.8 from normal levels (Figure 1).

**Figure 4:**
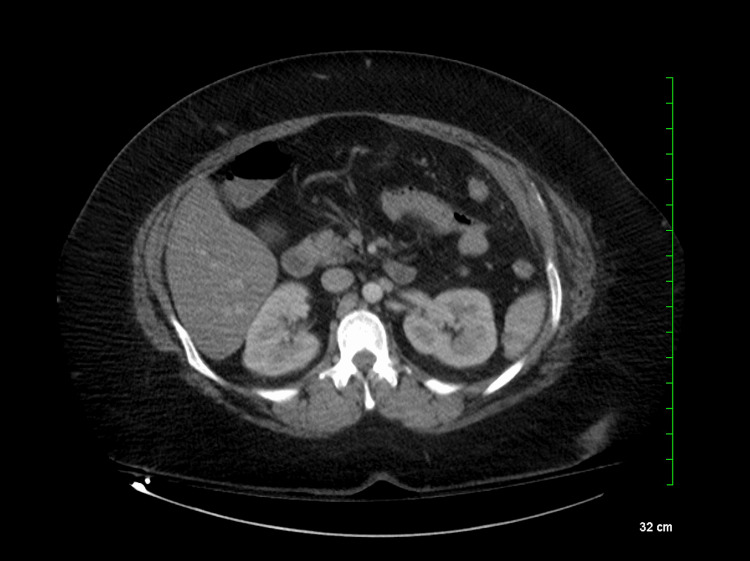
Normal kidneys No focal suspicious lesion or obstructive uropathy was evident.

A CT scan confirmed no remarkable anatomical issues with the kidneys (Figure 4). Given this finding, nephrologists recommended reducing IV fluid rate, maintaining a nil per os (NPO) status, avoiding nephrotoxic medications, adjusting piperacillin-tazobactam dosage, swapping IV vancomycin for IV daptomycin, and monitoring urinary sodium. Bumetanide was added to enhance urine output. Hemodialysis was initiated due to a lack of renal function improvement. The nephrologist suggested the acute tubular necrosis (ATN) was likely due to underlying sepsis and contrast toxicity. 

**Figure 5:**
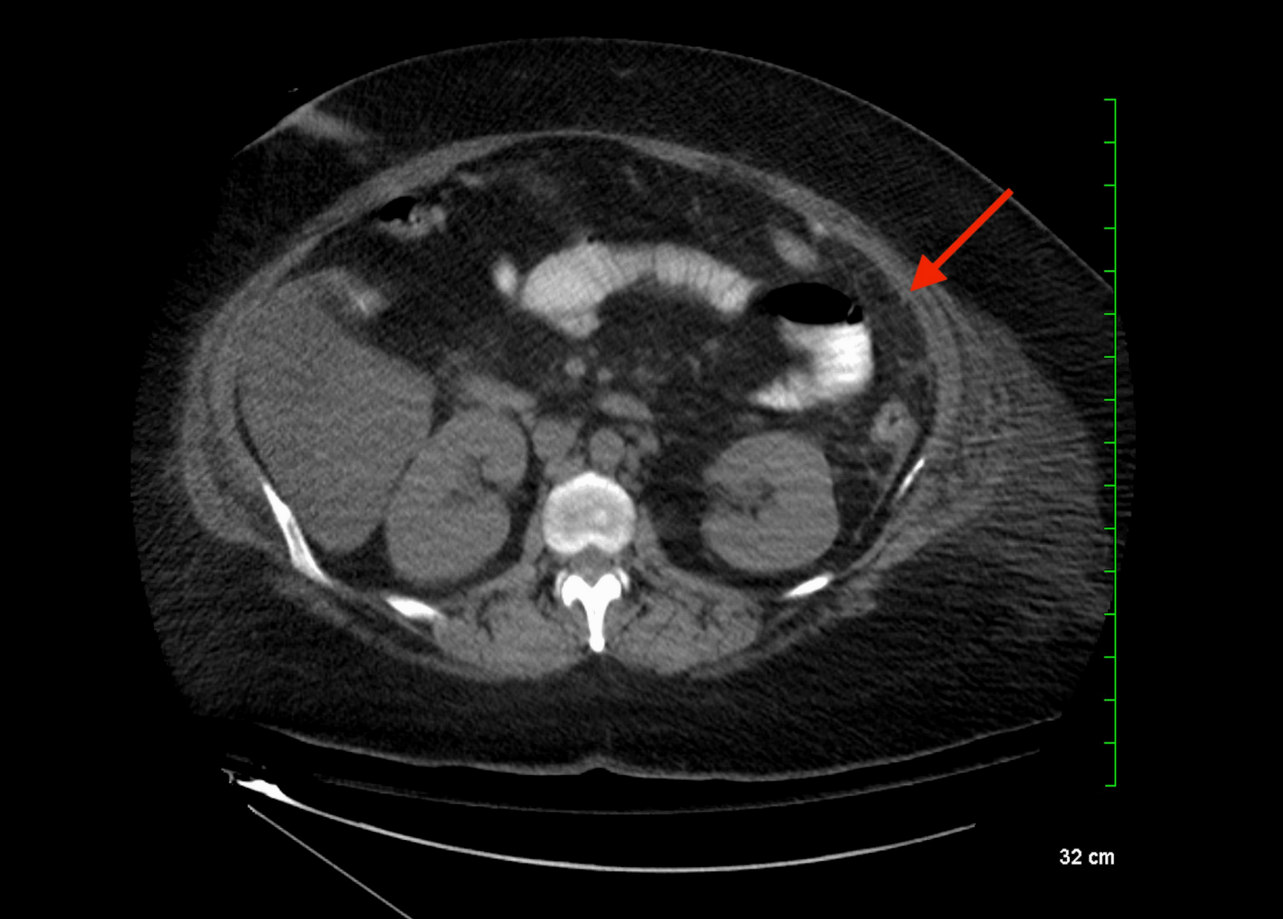
Resolution of previously identified left mid-abdominal abscess

On the fourth day, a follow-up CT scan showed the resolution of the abscess (Figure 5), though the patient continued to experience renal failure.

Despite hemodialysis, BUN and creatinine levels rose, and the patient had a low-grade fever and leukocytosis. Blood cultures indicated contamination with *Staphylococcus hominis* OXA-S. Operative cultures grew *Streptococcus viridans*, *Staphylococcus epidermidis*, and *Streptococcus anginosus*.

Improvement in urine output was noted by the seventh day. By the eighth day, renal recovery was evident, and the patient was moved from the ICU. However, intermittent fever and abdominal pain continued, requiring ongoing IV antibiotics, although no focal infections were found on a CT scan.

A permanent dialysis line was inserted on the 14th day due to persistent kidney issues, and antibiotics were discontinued as the patient stabilized. The patient was discharged on the 17th day after a challenging recovery, highlighting the importance of vigilant monitoring and multidisciplinary care.

## Discussion

Post-sleeve surgery, abscess formation is a possible complication that can present after the procedure [5]. Patients typically present with abdominal pain, tachycardia, leukocytosis, and fever postoperatively [5]. The most common site for these abscesses is the left upper quadrant (LUQ), particularly affecting the spleen [5]. A gastric leak caused by a failure of the staple line to completely close is linked to their formation and typically presents the first few days after the surgery [5].

According to prior studies, it has been shown that a strong risk factor for the development of an abscess is the presence of a patient with weakened immunity [6]. Other risk factors that predispose the patient to abscess formation include smoking, peptic ulcer diseases, co-existence of hiatal hernia, and hypercholesterolemia [9]. Smoking is strongly correlated with abscess formation, followed by hypercholesterolemia [9]. A study concluded that tobacco smoking two months before the surgical procedure corresponds with a higher risk of early complications, including abscess formation [9]. 

When a patient presents with symptoms suspicious of an abscess formation, the best diagnostic tool is an abdominal CT scan with contrast [7]. This form of imaging also helps to rule out the presence of a gastric leak [7]. An abscess on a CT scan shows obliteration of the fat plane and enhancement in the abscess wall [10]. If a gastric leak is suspected on a CT scan, findings such as free air under the diaphragm, elevated left diaphragm, and left pleural effusion would be seen [11]. Management of these abscesses varies based on the type of patient presentation [7]. If the abscess is small and non-loculated, it can be treated with antibiotics; however, if it is large, a percutaneous or laparoscopic drainage may be necessary [7].

To choose the most appropriate antibiotics, knowing the most common organisms present in these abscesses is the key. A study that followed 31 patients with post-laparoscopic sleeve gastrectomy leakage concluded that the most common microbial cultures included *Candida* species as well as other bacteria like *Klebsiella*, *Streptococcus*, and *Pseudomonas* [12]. To prevent infections from occurring, the first-line antibiotic that is given is cefazolin, with an alternative being cefotaxime or ceftriaxone [13]. If the patient has a history of IgE-mediated penicillin/cephalosporin allergy, then other antibiotics can be used like clindamycin plus a fluoroquinolone, clindamycin plus an aminoglycoside, or clindamycin plus aztreonam [13]. Common antibiotics used for treatment are subdivided into mild-moderate or severe infections [14]. For mild-moderate infections, the preferred options are ertapenem 1 g IV q24h or moxifloxacin 400 mg IV/PO q24h [14]. For severe infections, the preferred option is piperacillin/tazobactam 3.375 g IV q6h or 4.5 g IV q8h [14].

In the OR, a fair amount of adhesions were noted with a foul-smelling fluid. Cultures were then taken irrigated with antibiotics, and the abscess was noted to be in an unusual placement near the left colon. Abscesses caused by a sleeve gastrectomy directly are usually seen in the LUQ [5]. This suggests that the cause of the abscess was not due to surgical trauma or a staple leak but potentially due to a bacterial infection. 

Postoperatively, the patient was transferred to the ICU for symptoms of agitation and restlessness. Her symptoms were caused by the presence of an infection and AKI. The AKI was most likely caused by the presence of sepsis that this patient developed [15]. She was started on IV fluconazole at 400 mg daily to treat a potential *Candida* infection. She was concurrently on IV vancomycin and piperacillin and tazobactam until blood culture results were obtained. The patient’s creatinine concentration continued to rise while the WBC count decreased. The nephrology team recommended that this patient’s IV fluids be reduced, IV vancomycin be switched to IV daptomycin, and her urinary sodium be monitored. Operative culture results showed the presence of *S. anginosus* group organisms, which are common with abscess formation [12]. By day 8, the patient showed signs of renal recovery and was considered stable enough for a downgrade from the ICU. The patient was later administered permanent dialysis and able to be discharged by day seventeen.

The appearance of an abscess near the left colon following a sleeve gastrectomy is rare, leading us to conclude that the presence of this abscess was most likely caused by an issue with wound healing. According to a previous study conducted, this patient’s history of smoking and vaping predisposes her to impaired wound healing and, thus, more prone to abscess formation [16]. Although more research is warranted for the management and prompt identification of abscesses at atypical sites following sleeve gastrectomy, this case aims to highlight the importance of a comprehensive workup in patients with abdominal pain and a recent history of abdominal surgery.

## Conclusions

As sleeve gastrectomy becomes more common, addressing potential postoperative complications is essential. In this case, the abscess was determined to be unrelated to the procedure, with *S. anginosus* identified as the causative organism. Prompt surgical exploration, drainage, and antibiotic therapy were effective in managing the abscess. To reduce the risk of such complications, preventive measures like extended postoperative monitoring, early antibiotic administration, adequate IV fluids, parenteral nutrition, and early ambulation should be considered. Continued research into optimizing postoperative care protocols can help minimize complications in bariatric surgery.
